# Supplementary material for: Knowledge, perceptions and practices on antibiotic use among Sri Lankan doctors
Source: PLoS One. 2022 Feb 8;17(2):e0263167. doi: 10.1371/journal.pone.0263167 (PMC8824337; doi:10.1371/journal.pone.0263167)
Supplement: S1 Table — (DOCX) [file pone.0263167.s001.docx]

**S1 Table: : Marks allocation for knowledge scores**

|  | **Yes** | **No** | **Don’t know** |
| --- | --- | --- | --- |
| Antibiotics are used to prevent development of bacterial infections | 1 | 0 | 0 |
| Administration of antibiotics within one hour to a patient with sepsis is life-saving | 1 | 0 | 0 |
| Antibiotics should be prescribed to any patient with fever | 0 | 1 | 0 |
| Empirical antibiotic treatment needs to be deescalated or escalated according to microbiological culture results | 1 | 0 | 0 |
| Antibiotic dose needs to be decided according to age and body weight of the patient | 1 | 0 | 0 |
| Any antibiotic can be used to treat any bacterial infection | 0 | 1 | 0 |
| Antibiotic combinations are always better than monotherapy | 0 | 1 | 0 |
| All bacterial infections can be treated with same duration of antibiotics | 0 | 1 | 0 |

|  | **Correct choice** | **Correct choice + others** | **Incorrect choice** |
| --- | --- | --- | --- |
| Which of the following antibiotic/s belongs to beta lactam group? | 0.25 each | _ | - 0.25 each |
| Cause/s of Antimicrobial Resistance (AMR) development and spread, | 0.25 each | _ | - 0.25 each |
| Select the drug treatment option which is most favourable to treat patients who are positive with following resistant phenotypes. [MRSA] | 1 | 0 | 0 |
| Select the drug treatment option which is most favourable to treat patients who are positive with following resistant phenotypes. [CRE] | 1 | 0 | 0 |
| Select the drug treatment option which is most favourable to treat patients who are positive with following resistant phenotypes. [ESBL] | 1 | 0 | 0 |
| Select the drug treatment option which is most favourable to treat patients who are positive with following resistant phenotypes. [VRE] | 1 | 0 | 0 |
