# Supplementary material for: Knowledge, perceptions and practices on antibiotic use among Sri Lankan doctors
Source: PLoS One. 2022 Feb 8;17(2):e0263167. doi: 10.1371/journal.pone.0263167 (PMC8824337; doi:10.1371/journal.pone.0263167)
Supplement: S2 Table — (DOCX) [file pone.0263167.s002.docx]

**S2 Table: Marks allocation for practice scores**

|  | **Almost always/ Often** | **Sometimes** | **Rarely/ Never** |
| --- | --- | --- | --- |
| Do patients demand antibiotics from you? | 0 | 1 | 2 |
| Do you feel under pressure if your patient expects an antibiotic prescription? | 0 | 1 | 2 |
| Do you select antibiotics according to local/ international guidelines? | 2 | 1 | 0 |
| Do you refer BNF (British National Formulary) when prescribing antibiotics? | 2 | 1 | 0 |
| Do you order cultures before commencing antibiotics in your hospital practice? | 2 | 1 | 0 |
| Do you request for cultures before commencing antibiotics in your private practice? | 2 | 1 | 0 |

|  | **Yes** | **No** | **Unanswered** |
| --- | --- | --- | --- |
| Do you consider AMR when prescribing? | 1 | 0 | 0 |
| Do you practice de-escalation therapy? | 1 | 0 | 0 |
| Do you practice IV to oral switch? | 1 | 0 | 0 |

| How do you decide the dose of antibiotics, route, frequency of administration?* | **Choices** | **Marks** |
| --- | --- | --- |
|  | By remembering | 0 |
|  | By referring to BNF or another formulary ± other choices | 2 |
|  | By following my seniors’ practice/ By following my colleagues or both | 1 |

*The total mark achievable is 2
