# Supplementary material for: Knowledge, perceptions and practices on antibiotic use among Sri Lankan doctors
Source: PLoS One. 2022 Feb 8;17(2):e0263167. doi: 10.1371/journal.pone.0263167 (PMC8824337; doi:10.1371/journal.pone.0263167)
Supplement: Attachment — Submitted filename: 03 01 2021 Point by point rebuttal.docx [file pone.0263167.s006.docx]

**Author response to reviewer comments**

**Comments to the Author**


PLOS ONE does not copyedit accepted manuscripts, so the language in submitted articles must be clear, correct, and unambiguous. Any typographical or grammatical errors should be corrected at revision, so please note any specific errors here.

Reviewer #1: No

**Author response: We have corrected the manuscript and hope the language it clearer now**

Reviewer #3: Yes

6. Review Comments to the Author

Please use the space provided to explain your answers to the questions above. You may also include additional comments for the author, including concerns about dual publication, research ethics, or publication ethics. (Please upload your review as an attachment if it exceeds 20,000 characters)

Reviewer #1: Authors are advised to address all of the comments. The write up of the manuscript still requires language editing.

**Author response: We could not find any reviewer comments to address. We had given our responses to the reviewer comments in our previous revisions, we would be most grateful if the reviewer can specify which comments need further addressing.**

**Thank you for suggesting language edits, we have attended to it. We hope the reviewer and the editors are satisfied with the changes made and look forward to any specific comments that may still need corrections.**

Reviewer #3: The authors have answered to all the queries made by the reviewers. The manuscript now in a better form and ready to be accepted.
