# Supplementary material for: Knowledge, perceptions and practices on antibiotic use among Sri Lankan doctors
Source: PLoS One. 2022 Feb 8;17(2):e0263167. doi: 10.1371/journal.pone.0263167 (PMC8824337; doi:10.1371/journal.pone.0263167)
Supplement: Attachment — Submitted filename: Reviewer Comment.doc [file pone.0263167.s004.doc]

PONE-D-21-34680

Research Article

Knowledge and practices on antibiotic use among Sri Lankan doctors

**Reviewer’s Comment**

**General Comments**: The manuscript requires copyediting for both content and English language.

**Specific Comments:**

**Abstract:**

**Introduction:** It would be better to avoid using abbreviations in abstract section. If it is used, follow only the internationally adopted abbreviations. I also suggest the author to use the term ‘antimicrobial’ rather than antibiotic.

**Methods:** The information provided in method section lack vital information about how the questionnaire was developed? Who were the study participants? How questionnaire was applied? How validation of the questionnaire was performed? How the scoring was executed? How validated response can be obtained through google form? Likert scale questions are generally used to assess the attitude of the respondents, but here it has been used for knowledge/practice?

**Key words**: The authors should make sure the keywords are MeSH words, particularly knowledge and practices,

**Main body:**

**Introduction**: Reorganize this section in different paragraph with background information, problem statement, rationale of the study

**Methodology**: It will be worthwhile mentioning the qualification and experiences of the experts who were involved in validation of the questionnaire. How reliability of the questionnaire was performed? Why different scoring was used, e.g. 0.25 score, 1 score, negative score, 0 score, 2 score? These are too confusing! The term ‘mark/s’ should better be replaced with the term ‘score/s’.

The information provided in method section lack vital information about how the questionnaire was developed? Who were the study participants? How questionnaire was applied? One respondent can fill the google questionnaire multiple times. How validated response can be obtained through google form? Likert scale questions are generally used to assess the attitude of the respondents, but here it has been used for knowledge/practice? Duration and location of the study?

**Results**: the number of unanswered responses are seen in few cases only, it would be better to merge them as ‘Don’t know/Unanswered’(table 2). Why the heading for Table 4 is below the table? Full form of the abbreviations used in table 4 should be mentioned in footer of the table. Table 6 is about the perception on antibiotic use, whereas there is no any word about perception in the title of the manuscript? It is quite unusual to use 4 points likert scale (table 6). It can be made into 3 scales (agree, Neutral/unanswered, disagree).

Practice can better be determined through observation of real prescriptions (table 7) not through asking the questions. The responses for this section through questionnaire particularly online will be biases-how many will say that they do malpractice???

It would be meaningful to use the median score rather than mean score.

There are nine tables in this manuscript. It is advisable to reduce the number of tables if feasible through merging or modifying the tables.

**Discussion**: Discussion require more comparisons and clarifications. Study limitations and recommendations are vital components of a manuscript.

**Conclusions:** This section should be short and conclusive with key message from the findings.

**References**: Make sure the references and their citations in the text are as per the journal’s requirements.
